# Nationally and locally-initiated One Health approach in controlling rabies in West Kalimantan, Indonesia

**DOI:** 10.14202/vetworld.2022.2953-2961

**Published:** 2022-12-28

**Authors:** Cut Desna Aptriana, Etih Sudarnika, Chaerul Basri

**Affiliations:** 1 Directorate of Veterinary Public Health, Ministry of Agriculture Republic Indonesia, Jakarta 12550, Indonesia; 2 Student in Majoring Veterinary Public Health, Postgraduate School, IPB University, Bogor 16680, Indonesia; 3 Department of Animal Disease and Veterinary Public Health, Veterinary and Biomedical School, IPB University, Bogor 16680, Indonesia

**Keywords:** infectious disease, One Health, rabies, zoonoses

## Abstract

**Background and Aim:**

Rabies is one of the prioritized zoonoses in Indonesia and West Kalimantan is one of the rabies-endemic provinces in the country. This study aimed to evaluate a locally-initiated One Health approach to implement rabies prevention and control programs in Pontianak City and Sanggau District (through a bottom-up approach), and the central government initiated a program in Ketapang District (through a top-down approach).

**Materials and Methods:**

Data were collected using three focused group discussions involving public health and animal health/veterinary sectors from each district or city. This study collected data from the rabies control program in West Kalimantan from 2014 to 2020.

**Results:**

The evaluation results of the rabies prevention and control program in Pontianak City and Sanggau District that used the local initiative approach were considered effective in reducing the number of rabies cases in these areas, and they overcame the challenges, for example, limited resources, in this area. Pontianak City and Sanggau District initiatives’ approach was a bottom-up policy. Thus, this program had better sustainability than the One Health approach in the Ketapang District, which used a top-down implementation. The approach in Ketapang District was also considered adequate to reduce the number of rabies cases in the area. However, the reshuffle of animal health officers and health workers in 2020, which was not followed by training on One Health for the new officers, became a challenge in implementing One Health in Ketapang District.

**Conclusion:**

National and local initiatives’ One Health approach implemented by Ketapang District, Sanggau District, and Pontianak City involved multiple sectors and was considered effective in preventing and controlling rabies in these areas. However, the sustainability of this program in the Ketapang District requires commitment and support from the local government.

## Introduction

Rabies is a viral disease that affects the central nervous system of mammals, including humans. The fatality rate is almost 100% in humans and animals; thus, rabies remains a global threat [1]. Rabies is one of the prioritized zoonoses in Indonesia. Rabies has been endemic in 26 of Indonesia’s 34 provinces, with an average of 80,861 cases of transmitting dog bites and 105 human death cases annually [2]. Rabies has been endemic in Kalimantan since 1974, with the first case in East Kalimantan. Subsequently, three cases have been reported: In Central Kalimantan in 1978, in South Kalimantan in 1983, and in West Kalimantan in 2005. West Kalimantan eliminated rabies in August 2014 [3]. In October 2014, the first case of rabies confirmed in Ketapang District returned West Kalimantan to the list of rabies-infected areas. Rabies cases are widespread in the province and 13 of the 14 districts have identified dogs with rabies [3, 4].

The challenges to be managed by the Provincial Government of West Kalimantan in preventing and controlling rabies include the large size of the area with limited human resources, budget, and animal health service facilities. Thus, an effective strategy is urgently required. The Provincial Government of West Kalimantan has used the One Health approach in implementing its efforts to prevent and control rabies and employed the local government’s policy. The One Health approach is a collaborative, multi-sectoral, and interdisciplinary approach that involves the public health, animal health, and environmental sectors [5]. The central government drafted a plan and implemented the top-down One Health approach in the region. Ketapang District has become a pilot location for implementing the rabies control program using the One Health approach in Indonesia. Using this approach (i.e., information sharing, integrated collaborative case investigation, and communication network in rabies cases in Ketapang District), rabies cases can be followed up in multiple sectors. West Kalimantan has implemented several local interventions based on the characteristics and initiatives of the target region, for example, the bottom-up interventions in Pontianak City and Sanggau District and the top-down policy in Ketapang District. A local initiative intervention is a policy issued by the district or city government that considers the limited local resources and rabies status in the area. For example, Pontianak City implemented a free rabies vaccination program for dogs on every Thursday called ASIKIN (Ayo Vaksinasi Rabies Kamis Ini; in English, the meaning is Let’s get rabies vaccinated on Thursday), and DOKELING (Dokter Hewan Keliling; in English, the meaning is mobile veterinarian services), mobile veterinarian services for the area surrounding the city. Sanggau District implemented a local initiative approach called SABER 24 (Sanggau Bebas Rabies Tahun 2024; in English, the meaning is Sanggau rabies-free in 2024), supported by the central and provincial governments.

Pontianak City, Sanggau District, and Ketapang District were selected as the research objects for this study because they combined the national and local initiatives implemented in the One Health approach to rabies prevention and control programs. This study aimed to evaluate the program implemented in these three locations from 2014 to 2020.

## Materials and Methods

### Ethical approval

The Human Research Ethics Committee of IPB University approved the ethical clearance and informed consent for this study: 695/IT3.KEPMSM-IPB/SK/2022.

### Study period and location

The study was conducted on January 20–21, 2022, in Pontianak City, West Kalimantan, Indonesia. This study used three interventions as case studies to assess rabies control programs in West Kalimantan Province using the One Health approach.

### Data collection

Data were collected using three focus group discussions (FGDs). The participants in the FGDs were animal and public health officers in Pontianak City, Sanggau District, and Ketapang District. The FGDs were held at a hotel in Pontianak City. Specifically, the 20 participants in FGDs were as follows: Three representatives of the Ministry of Agriculture, the head of the West Kalimantan provincial animal health office, one representative of the head of the animal health office of Ketapang District, one representative of the head of the Sanggau District animal health office, the head of the Pontianak City office, representatives of the rabies program of the animal health office of West Kalimantan, one representative of the rabies program of Ketapang District, one representative of the rabies program of Sanggau District, one representative of the rabies program of Pontianak City, the head of the provincial health office of West Kalimantan, one representative of the head of Ketapang District health office, one representative of the head of the Sanggau District health office, one representative of the head of the Pontianak City health service, two representatives of the West Kalimantan provincial health office rabies program, one representative of the Ketapang District rabies program, one representative of the Sanggau District rabies program, and one representative of the Pontianak City rabies program.

The selection of participants was conducted by the head of the animal health office and the health office related to their duties and authorities in the rabies control program in West Kalimantan Province, Ketapang District, Pontianak City, and Sanggau District. The three moderators of the FGDs held a One Health master trainer certificate organized by Food and Agriculture Organization (FAO) and the Ministry of Agriculture. The language used during the FGDs was Indonesian.

Of the three FGDs, the first was conducted to collect data related to the identification of challenges in the rabies control program in Pontianak City, Sanggau District, and Ketapang District; the second was conducted to collect data related to rabies cases in these three areas; and the third was related to the cooperation across sectors in implementing the rabies control programs in the three areas. Data collection instruments were as follows: The FGDs’ questionnaire guidelines, an audio recorder, a laptop, a camera, and stationery.

### Statistical analysis

Data were analyzed using the content analysis approach by identifying FGD results with Microsoft Excel 2016 (Microsoft, Washington, USA).

## Results

**Figure-1:**
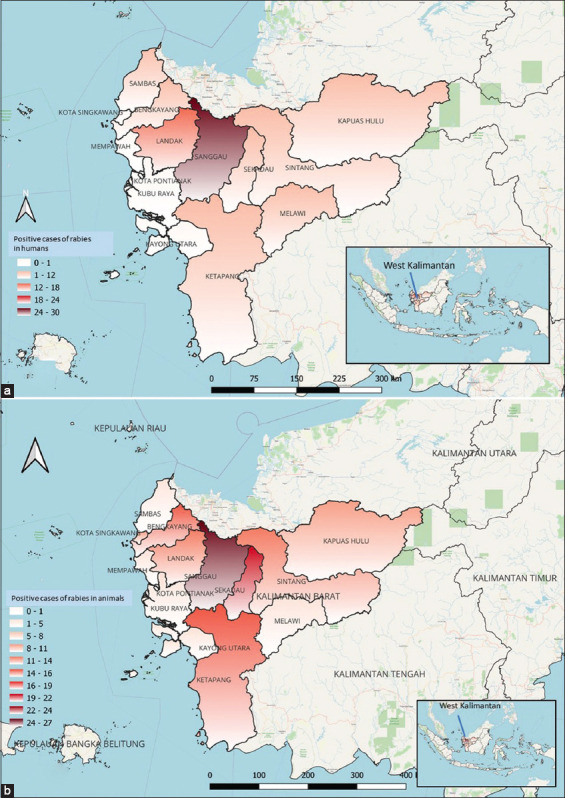
Rabies Case Distribution in West Kalimantan from 2014 to 2020: (a) Rabies cases, humans and (b) rabies cases, animals [Source: Map generated by QGIS 3.22.5 software].

West Kalimantan shares borders with Central Kalimantan, Sarawak of East Malaysia, and East Kalimantan. A challenge for the province preventing and controlling rabies (Figure-1) is those bordering provinces in Malaysia are rabies-infected. In addition to sharing borders with infected areas, the province is 146.807 sq. km, and thus requires many animal health officers and service facilities to serve all the villages. Access to these remote locations is difficult and presents an additional set of challenges because most of these places are among mountains with hundreds of rivers.

The national strategy for preventing and controlling rabies in the infected areas comprise vaccination of rabies for dogs, information distribution, implementation of Integrated Bite Case Management (IBCM), surveillance and dog population management (DPM), animal movement monitoring, and investigation. For rabies-free or rabies-susceptible areas, the strategy comprises vaccination, information distribution, implementation of IBCM, animal movement monitoring, and DPM [6]. The head of the West Kalimantan provincial animal health officials said that obstacles in detecting rabies cases in dogs in the three locations were usually caused by losing track of the dog(s) or the dogs being killed by the community without reporting the case(s) to animal health officers.

### One Health approach in Ketapang district

The rabies prevention and control program implemented in Ketapang District from 2014 to 2020 is shown in Table-1. Budget support for this program was from the central, provincial, and district governments. There was also intervention support from the FAO in 2017. This intervention included training for public health and animal health officers, which serves as the forerunner for improved implementation of One Health in the district.

**Table-1:** Rabies prevention and control program in Pontianak City, Sanggau District, and Ketapang District From 2014 to 2020

Year	District/city	Funding Source				Activity							
	
		Central Government	Provincial Government	District/city government	FAO/others	Vac	Inf	Tra	Eli	RT	Inv	PrPP	PEP
2014	Pontianak	-	-	v	-	-	-	v	-	-	-	-	-
	Sanggau	-	-	-	-	-	-	-	-	-	-	-	-
	Ketapang	v	v	v	-	v	-	-	v	-	-	v	v
2015	Pontianak	-	-	-	-	-	-	-	-	-	-	-	-
	Sanggau	-	-	-	-	-	-	-	-	-	-	-	-
	Ketapang	v	v	v	-	v	v	-	v	v	-	v	v
2016	Pontianak	-	-	-	-	-	-	-	-	-	-	-	-
	Sanggau	v	v	v	-	v	v	-	-	-	-	v	v
	Ketapang	v	v	v	-	v	v	v	v	v	-	v	v
2017	Pontianak	-	v	v	-	-	v	-	-	-	-	-	-
	Sanggau	-	v	v	-	v	v	-	-	-	-	v	v
	Ketapang	v	v	v	v	v	v	v	v	v	v	v	v
2018	Pontianak	-	v	v	-	v	v	-	-	-	-	v	v
	Sanggau	-	v	v	-	v	v	-	-	-	-	v	v
	Ketapang	v	v	v	v	v	v	v	v	v	-	v	v
2019	Pontianak	-	v	v	-	v	v	-	-	-	v	v	v
	Sanggau	-	v	v	v	v	v	-	-	-	-	v	v
	Ketapang	v	v	v	v	v	v	v	v	-	v	v	v
2020	Pontianak	-	v	v	-	v	v	-	-	-	v	v	v
	Sanggau	-	v	v	v	v	v	-	-	-	v	v	v
	Ketapang	-	v	v	-	v	v	-	v	-	v	v	v

Vac=Vaccination, Inf=Information Distribution, Tra=Training, Eli=Elimination, RT=Rapid Test of Rabies-Carrier Animals, Inv=Investigation, PrPP=Pre-exposure prophylaxis, PEP=Post-exposure prophylaxis

Implementing the rabies control program with One Health in Ketapang District triggered human and animal health officers to respond to and report bite cases from the community, reducing the limitation between animal and human health resources, reducing operational costs of investigations, and making rabies cross-sectoral collaboration.

**Figure-2:**
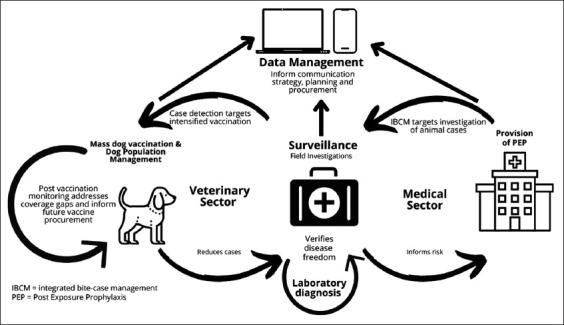
Illustration of One Health scheme for rabies prevention and control program [7].

The implementation of One Health in the Ketapang District proceeded as planned (Figure-2) [7]. The community reported the bite case to the public health authority and then coordinated, communicated, and collaborated with the animal health officer. The animal health sector performed case detection of rabies-suspected biting dogs, which were observed and tested in the laboratory to confirm the diagnosis. The animal health and public health officers used communication networks through phone calls, communication applications with a unique reporting format, and reporting on the Sistem Informasi Zoonosis dan EID (SIZE 2.0) platform. SIZE 2.0 is a health surveillance information system that links the three other systems; 1) Ministry of Health’s Sistem Kewaspadaan Dini dan Respon – SKDR (Early Warning Alert and Response System – EWARS), 2) Ministry of Agriculture’s Integrasi Sistem Informasi Kesehatan Hewan Nasional (Integrated National Animal Health Information System – iSIKHNAS), and 3) Ministry of Environment and Forestry’s Sistem Informasi Kesehatan Satwa Liar (Wildlife Health Information System – Sehat Satli) [8].

Implementing the One Health approach decreased rabies cases in Ketapang District. The human case fatality rate in Ketapang District was 6.25% in 2014, 1.03% in 2015, and zero in 2019 (no death cases). However, rabies incidence increased to 1.75 cases per 1 million population per year in 2020, and the number of human deaths increased to 0.84% (Table-2). The reshuffle of animal health officers and health workers in 2020, which was not followed by training on One Health for the new officers, became a challenge in implementing One Health in Ketapang District. The impact of the reshuffle also led to an increase in rabies cases in 2020 in Ketapang District. In addition to the positive impact on reducing the number of rabies cases, the One Health approach has had a positive effect on animal vaccination activities and improved information distribution. Based on the results of the FGD, the active participation of the community in vaccination and case reporting in the Ketapang District is one of the positive impacts of implementing the One Health approach program.

**Table-2:** Rabies cases in Pontianak City, Sanggau District, and Ketapang District from 2014 to 2020.

Year	Regency/Municipality								

	Pontianak (Status: Rabies-free)			Sanggau (Status: Infected)			Ketapang (Status: Infected)		
		
	Bite case (case/ 1 million population - year)	Rabies- caused human Fatality (CFR%)	Rabies incidence (case/1 million population -year)	Bite case (case/ 1 million population - year)	Rabies - caused human Fatality (CFR%)	Rabies incidence (case/1 million population - year)	Bite case (case/ 1 million population - year)	Rabies- caused human Fatality (CFR%)	Rabies incidence (case/1 million population - year)
2014	0	0	0	0	0	0	206,79	6,25	23,69
2015	0	0	0	33,74	0	0	405,48	1,03	8,4
2016	0	0	0	296,98	4,48	17,73	288,59	0	6,18
2017	90,91	0	0	2.604,32	0,92	43,69	317,12	0	6,05
2018	36,07	0	0	2.347,01	0,46	19,39	339,28	0,58	3,96
2019	88,15	0	0	2.928,39	0,58	34,02	680,59	0	0
2020	57,69	0	0	1.744,92	0	6,18	208,53	0,84	1,75

GHPR=*Gigitan Hewan Penular Rabies* (rabies-carrier animal bite), CFR=Case Fatality Rate

### Local initiative one health approach in Sanggau district

In contrast to Ketapang District, the rabies prevention and control program in Sanggau District was based on local government initiatives. The program was implemented from 2016 to 2020 with support from central, provincial, and district governments (Table-1).

The challenges experienced by the district government were limited funds for the rabies control program, limited veterinarians at the animal health office, limited animal health service facilities, and that most of the dogs in Sanggau District were not caged. These challenges caused the Sanggau District government to set a policy that was a local initiative of the Sanggau District Government. In 2019, the Sanggau District worked with local plantations companies in Sanggau District to use their corporate social responsibility funds to support vaccination activities for rabies-carrier animals and information distribution on rabies to residents in the area around the plantation companies.

The number of bite cases and human death cases due to rabies in Sanggau District from 2015 to 2019 caused the local government to implement their local initiative approach, SABER 24, Sanggau Bebas Rabies Tahun 2024 (Sanggau Free of Rabies in 2024). This program included the integrated, massive, and simultaneous dog vaccination that involved plantation companies and other sectors. The cross-sectoral involvement in SABER 24 increased the effectiveness of the rabies prevention and control program in Sanggau District more effective, particularly for the vaccination of rabies-carrier animals and information distribution activities.

As shown in Figure-2, the implementation of cross-sectoral collaboration in SABER 24 was similar to the scheme in Ketapang District. This integrated investigation from the public health and animal health sectors also resulted in the use of appropriate post-exposure prophylaxis (PEP) according to the needs of the bite case. The number of dog bite cases in Sanggau District was the highest in West Kalimantan Province from 2017 (2604.32 cases per 1 million population per year) to 2019 (2928.39 cases per one million population per year) (Table-2).

SABER 24, conducted in Sanggau District, had no special training activities for animal health and public health officers. These officers coordinated, communicated, and collaborated cross-sectorally by optimizing the existing communication network, namely, GHPRS Sanggau. Collaboration was also conducted in case investigation activities and integrated rabies socialization.

There was no specific platform for reporting systems related to rabies cases in Sanggau District. Rabies case reporting data were recorded in each sector and then submitted to the SABER 24 meeting forum for clarification and confirmation of the data. Efforts to prevent and control the rabies program in Sanggau District also involved the Indonesia Armed Forces-Indonesia National Police and extension officers from Agricultural Offices and plantation companies.

### Locally initiated one health approach in Pontianak city

The City of Pontianak was the only area rabies-free area in West Kalimantan Province. Similar to Sanggau District, Pontianak City implemented local initiatives. This city had a relatively more sufficient number of veterinarians than other areas because the dog-keeping system of the former was more systematic than that of the latter, with the former having pets housed in cages. The residents of Pontianak City also participated actively in vaccinating their rabies-carrier animals and actively reported bite cases. Thus, Pontianak City’s government was able to implement the rabies prevention and control program well.

Pontianak City’s limitations and challenges were the shared border with the District of Mempawah, a rabies-infected district with a rabies incidence of 3.31 cases per 1 million population per year, and the District of Kubu Raya with an incidence of 0.50 cases per 1 million population per year. The absence of special-allocated budget support for rabies forced the local government of Pontianak City to implement a local initiative. The budget support and rabies prevention and control program in Pontianak City are shown in Table-1.

The Mayor of Pontianak City issued a circular called Regulating the Rearing of Rabies-Carrier Animals in the City of Pontianak in 2017 [9]. This circular appealed to and encouraged residents to be alert when in rabies-infected areas and not to transport rabies-carrier animals, (e.g., dogs, cats, monkeys, and weasels) from such places into Pontianak City. To prevent the spread of the disease, Pontianak City must ensure that every dog participates in the mass vaccination performed by officers from the animal health office. In addition, residents must immediately report bite cases of dogs or other rabies-carrier animals that would lead to rabies.

This local initiative highlights the activities in the veterinary sector, such as vaccination and information distribution. Since 2018, the establishment of ASIKIN resulted in administering free vaccinations every Thursday for rabies-carrier animals, in the Animal Health Service Center (Pusat Kesehatan Hewan – Puskeswan) of Pontianak City. Another activity implemented was DOKELING, a mobile veterinary service using a car operated by veterinarians and para-veterinarians in Pontianak City. These activities helped the residents of Pontianak City vaccinate their pets for free and provide services for those who live far from veterinary service facilities. The City of Pontianak had a communication forum between their veterinary and public health sectors, as well as other sectors, in the form of The Rabies Center. The Rabies Center increased access to cross-sectoral communication and led to improved and additional integrated rabies-relevant activities, such as investigation and surveillance. The rabies center also involved other sectors in the community (the communication forum of local government and law enforcement) from sub-districts and villages in Pontianak City.

The responsibility to vaccinate rabies-carrier animals was that of the veterinary sector and public was responsible for providing PEP. These activities were in line with the illustration of the One Health implementation scheme (Figure-2). Every bite case was rapidly responded to by implementing IBCM by the public health sector and immediately coordinated with the veterinary sector to conduct detection of the biting animal for further observation. Coordination, communication, and collaboration performed by the local office in charge of animal health and the Pontianak City Health Office were running well. The coordination and communication on bite cases were performed through phone calls from the public health sector to the veterinary sector or vice versa.

Similar to the implementation initiative in Sanggau District, no special training was allocated for Pontianak’s veterinary and public health officers. The reporting system was also conducted without a specific platform. Each sector had its unique rabies-reporting system, shared and reported to other sectors in the cross-sectoral meeting in Pontianak City. Local initiatives implemented in Pontianak City resulted in no rabies-caused human death and no rabies incidence from 2014 to 2020. FGD participants said that the number of rabies bite cases in Pontianak City was lower than in other districts.

## Discussion

Rabies prevention and control programs were implemented efficiently using the One Health approach that involves cross-sectoral collaboration between animal, human, and environmental health [10]. The limitations and challenges experienced by district and city governments required initiatives in the policies that were increasingly directed at solving significant community challenges [11].

Strategic actions supported the success of rabies prevention and control programs with the One Health approach: Systematic data collection and compilation; coordination and sharing of data among the animal, human, and environmental health sectors; effective, efficient surveillance; availability and sufficiency of PEP; increased vaccination coverage and efficacy; increasing public awareness; increased laboratory capacity; and research activities [12].

Coordination and sharing of cross-sectoral data have been well-established in Ketapang District, Pontianak City, and Sanggau District. However, systematic data collection and compilation were hindered in Sanggau District and Pontianak City because no specific platform was provided for cross-sector joint data collection and compilation. In contrast with the other two districts, Ketapang District has a specific format for reporting cross-sectoral rabies cases. However, reporting is in the form of manual recording and not in the form of a computerized database system. In addition, the SIZE (health surveillance information system) platform is not fully operational at the local government level. Only the central government can access it.

The constraints in making effective policies in the three locations are also caused by the limited data owned by animal health and human health sectors related to human and dog population density, vaccination data in animals and humans, data on rabies cases in dogs, data on the use of PEP, and insufficient of comprehensive data management and analysis. The One Health approach works best with a regular reporting system that allows for evaluations of periodic program progress, disease status, and social-economic impacts. Information from a regular reporting system will also be beneficial in developing control strategies or programs at the national level [13]. The programs implemented in Sanggau District, Pontianak City, and Ketapang District optimized cross-sectoral surveillance. The lack of integrated surveillance was the primary constraint in understanding rabies dynamics locally and effectively controlling the disease [14].

Implementing cross-sectoral integrated surveillance can substantially improve case detection so that information on detected cases can impact the use of proper PEP [14]. Sufficiency and availability of PEP are the only prevention of rabies in humans after the bite of a rabies-carrier animal [12]. Rabies is fatal to humans when a human is bitten by a rabies-infected animal and is not immediately treated using PEP [15]. Rabies burden will be higher when PEP cannot be directly accessed after a bite incident by a rabies-suspected animal. The vast conditions of Sanggau District and Ketapang District had inaccessible topographical access, and limited health facility services made obtaining pre-exposure prophylaxis and PEP difficult. One of the best measures to prevent rabies-caused human fatality is to optimize the IBCM, which requires cross-sectoral rabies surveillance.

The community’s involvement in preventing and controlling rabies was also crucial in achieving the program’s success [6]. Local initiatives implemented by Sanggau District in establishing SABER 24 involved the community. SABER 24 involved the community in animal vaccination activities and information distribution. In addition, the community actively reported bite cases or animals suspected of having clinical symptoms of rabies to animal or human health officers. Increasing public awareness of the importance of vaccinating and responsibly caring for their rabies-carrier animals also helped reduce the number of rabies cases and human fatalities in Sanggau District.

The impacts of the SABER 24 program in Sanggau District were a decrease in human deaths in 2020 and an increase in the number of veterinarians and animal health officers in 2020. Sanggau District received an award from the Governor of West Kalimantan in 2021 for being the most committed district or city in controlling rabies. Similar to Sanggau District, Pontianak City, with the establishment of ASIKIN, and DOKELING, and the publishing of the Pontianak Mayor’s circular had a positive impact on the community regarding active participation in case reporting, vaccination of rabies-carrier animals, and responsible animal rearing. Those activities became the critical point of their success in preventing and controlling rabies in their city.

Local initiatives implemented by the city government of Pontianak have been considered adequate because the number of bite cases decreased from 88.15 cases per 1 million population per year in 2019 to 57.69 cases per 1 million population per year in 2020. Pontianak City will probably maintain its status as a rabies-free area in West Kalimantan with no cases of human death due to rabies and no cases of rabies. Local initiatives in Sanggau District and Pontianak City implemented policies using the bottom-up approach. This approach identifies the network of actors involved in providing services in one or more local areas [16] and progresses in line with the actions performed in the field. Although the coverage is at the district or city level, this approach is usually more transformative and responsive to the local social, environmental, and economic settings than the top-down approach [17]. This bottom-up policy approach is ongoing. Despite the limitations and challenges, the policy executors’ sense of ownership greatly substantially the program’s sustainability.

Unlike Sanggau District and Pontianak City, One Health in Ketapang District implemented a top-down approach. This top-down approach required explicit instruction from the central government and supervision in program implementation [18]. One benefit of this top-down approach was the central government’s financial support for capacity-building for veterinary and public health officers. However, this approach made obtaining commitments from activity executors difficult.

The change in structure of the local government became one of the challenges in implementing One Health activities. This challenge resulted in increased rabies bite cases and rabies incidents in Ketapang District. One Health trained veterinary and public health officers were moved or promoted to other positions or units. This personnel restructuring was not followed by capacity-building activities for the new personnel and presented a challenge in sustaining the rabies prevention and control program’s implementation. This requires commitment and support from the district government in the form of the legal framework and budget allocation to improve the officers’ performance to ensure the program is implemented.

The main limitation of this study was that the duration of the rabies prevention and control program in each district or city in this study differed and was relatively short; thus, the assessment of program sustainability in each district or city was limited. Thus, further research is necessary.

## Conclusion

Rabies prevention and control programs in Pontianak City and Sanggau District used locally initiated One Health approaches to effectively reduce the number of rabies cases in their respective areas. This type of bottom-up policy is required in responding to challenges, for example, limited resources. Local initiatives performed by the District Government of Sanggau and the City Government of Pontianak are ongoing. The sense of ownership embodied by the policy executors was one of the determining factors in the program’s sustainability. The top-down approach implemented by Ketapang District has also been proven effective in reducing the number of rabies cases. However, a strong commitment and support from the local government in the form of a legal framework and budget allocation to improve field officers’ performance to ensure the program’s sustainability and betterment must also occur.

## Authors’ Contributions

CDA and ES: Conceived and designed the study. CDA, ES, and CB: Analysis, interpretation of data, drafted, and revised the manuscript. All authors have read and approved the final manuscript.
